# First nationwide survey on *Pseudomonas aeruginosa* in Bolivia: susceptibility profiles, resistome, and genomic epidemiology

**DOI:** 10.1128/aac.01163-25

**Published:** 2025-11-11

**Authors:** Neisa Alvarado-Orosco, Maria A. Gomis-Font, Miquel A. Sastre-Femenia, Silvia Ribera Ortiz, Maritza Miranda Arce, María Elena Araúz Barba, María del Rosario Navia Durán, Sandra Grisel Vargas Nattez, Gabriela Cabellos Astorga, Paola Janeth Villarroel Rodriguez, Damariz Daniela Almaraz Roca, Marleni Castillo Cruz, Juan Pablo Chambi Condori, Pedro Quevedo Ribera, Suan Mishelly Patino García, Yenny Contreras Otalora, Flor María Rosales Rea, Claudia Milena Portugal Gutiérrez, Marisol Sánchez Serrano, Carla López-Causapé, Antonio Oliver

**Affiliations:** 1 UF of Bacteriology, National Center for Tropical Diseases (CENETROP) 279124 , Santa Cruz de la Sierra, Bolivia; 2 Department of Microbiology, Hospital Universitario Son Espases, Instituto de Investigación Sanitaria Islas Baleares (IdISBa), CIBERINFEC 375118, Palma, Spain; 3 Microbiology Laboratory, Hospital San Juan de Dios, Santa Cruz de la Sierra, Bolivia; 4 Department of Bacteriology, Hospital Universitario Japonés 58646 , Santa Cruz de la Sierra, Bolivia; 5 Microbiology Laboratory, Hospital Obrero N. 3, Santa Cruz de la Sierra, Bolivia; 6 Microbiology, Hospital Santa Bárbara, Sucre, Bolivia; 7 Microbiology Laboratory, Hospital del Norte, El Alto, La Paz, Bolivia; 8 Microbiology Laboratory, Hospital Clinico Viedma 279143 , Cochabamba, Bolivia; 9 Microbiology, Hospital Elizabeth Seton-Caja Petrolera de Salud, Cochabamba, Bolivia; 10 Department of Bacteriology, Instituto Gastroenterológico Boliviano-Japonés, Cochabamba, Bolivia; 11 Microbiology Laboratory, Hospital Daniel Bracamontes, Potosí, Bolivia; 12 Microbiology Laboratory, Hospital de Tercer Nivel, Montero, Santa Cruz de la Sierra, Bolivia; 13 Department of Bacteriology, Hospital Municipal Bajío del Oriente, Santa Cruz de la Sierra, Bolivia; 14 Microbiology Laboratory, Hospital Municipal Villa Primero de Mayo, Santa Cruz de la Sierra, Bolivia; 15 Microbiology Laboratory, Hospital Municipal Francés, Santa Cruz de la Sierra, Bolivia; 16 Department of Bacteriology, Hospital Municipal Alfonso Gumucio Reyes, Montero, Santa Cruz de la Sierra, Bolivia; 17 Laboratory HODE Materno Infantil-CNS 377468, La Paz, Bolivia; 18 Department of Bacteriology, Hospital Caja de Salud Caminos, Santa Cruz de la Sierra, Bolivia; Columbia University Irving Medical Center, New York, New York, USA

**Keywords:** genomic epidemiology, *Pseudomonas aeruginosa*, antimicrobial resistance

## Abstract

Information on the molecular epidemiology of *Pseudomonas aeruginosa* and antimicrobial resistance mechanisms is still limited in some South American countries. This study aims to decipher the population structure of 111 extensive drug-resistant *P. aeruginosa* isolates from a national study conducted in Bolivia during 2023–2024. The antibiotic susceptibility profiles were determined for 15 antipseudomonal agents. All isolates were subjected to whole-genome sequencing (WGS), and, through bioinformatics analysis, sequence types (ST), clonal relatedness, and acquired mutation-driven and transferable resistance mechanisms were elucidated. The most active antipseudomonal agents were colistin (98.2% intermediate, MIC_50/90_=1/2 mg/L) and cefiderocol (92.7% susceptible, MIC_50/90_=0.25/4 mg/L) according to the Clinical and Laboratory Standards Institute (CLSI). High resistance rates to ceftazidime/avibactam (79.3%), ceftolozane/tazobactam (82.9%), and imipenem/relebactam (71.2%) were documented. Carbapenemases were found in 60.3%, particularly including metallo-β-lactamases (MBL), such as SPM-1 (35%), VIM-2 (9%), the co-production of NDM-1 and DIM-1 (4%), or the new IMP variant IMP-111. Extended-spectrum β-lactamases (ESBLs) were detected in 12% of the isolates, including OXA-17 (7%), PER-1 (3%), and some GES variants. The most commonly detected clone was ST277 (35%) associated with SPM-1, followed by the ST309 (25%) producer of OXA-2 and various GES, and ST235 (20%) related with OXA-17 and new IMP-111. These clones harbored other acquired resistance genes, including emerging 16S rRNA methyltransferases, RmtD and RmtG. The high resistance rates for novel beta-lactams linked to an alarming spread of high-risk clones ST277 and ST235 and the very high prevalence of MBLs and ESBLs raise significant concern. This underscores the urgent need for establishing epidemiological surveillance and infection control strategies.

## INTRODUCTION

Antimicrobial resistance (AMR) is a major global threat (1) responsible for an estimated 1.27 million deaths in 2019 (2). This fact positions AMR as one of the most pressing challenges to global health in modern society, requiring coordinated action at all levels. This scenario in Latin America has worsened significantly, with carbapenem resistance among gram-negative bacteria increasing from 0.3% in 2002 to 21% in 2016. Some countries have reported prevalence as high as 50% according to the Latin American Network for Antimicrobial Resistance Surveillance (ReLAVRA) (3).

Although 30 out of 33 countries in Latin America and the Caribbean are developing or have completed national action plans to combat AMR, only 19 report to the ReLAVRA (4). Bolivia, one of the lowest-resourced South American countries, has scarce data on antibiotic resistance in bacterial pathogens. Nevertheless, this country stands out with the highest age-standardized AMR-related mortality rate globally, with 2,500 deaths attributable to AMR and 10,100 associated deaths reported in 2019 (2).

The global spread of the so-called ESKAPE pathogens, particularly *P. aeruginosa*, is a major cause of severe nosocomial infections, including ventilator-associated pneumonia and burn wound infections, both associated with high mortality rates (>30%) (5, 6). This threat rises from its ability to develop resistance to nearly all available antibiotics either by chromosomal mutations or through the horizontal acquisition of resistance genes (7–10), resulting in complex resistance profiles, such as those defined by the European Center for Disease Prevention and Control (ECDC) (multidrug resistance [MDR], extensive drug resistance [XDR], and pandrug resistance [PDR]) or the Infectious Diseases Society of America (IDSA) (difficult to treat resistance [DTR]) (11, 12). Additionally, *P. aeruginosa* has a nonclonal epidemic population structure with a few widespread high-risk MDR/XDR clones emerging from a diverse and highly recombining genetic background (13). Several studies have evidenced specific MDR/XDR *P. aeruginosa* strains, the so-called high-risk clones, which have been disseminated in hospitals worldwide causing multiple outbreaks (13, 14). Whole-genome sequencing (WGS) is providing valuable insights into the complex and dynamic resistome of MDR/XDR *P. aeruginosa* high-risk clones (10, 13, 15, 16).

Besides this alarming situation, the rise of carbapenem-resistant *P. aeruginosa* (CRPA) is concerning and led the World Health Organization to classify it as a high-priority pathogen in 2024 (17, 18). Although the emergence of carbapenemases has fueled the spread of carbapenem-resistant Enterobacterales, the extent to which carbapenemases contribute to CRPA appears to be more variable (19). In a recent global cohort study, KPC-2 and VIM-2 were the most commonly detected carbapenemases, particularly in Central and South America (20). Previously, a global surveillance study conducted across 12 countries, including isolates from South America, detected VIM and GES in nearly one-third of the isolates (21). A recent study from Brazil found that 55% of the isolates carried carbapenemases, with a notable increase in the prevalence of NDM (22). Moreover, neighboring countries, such as Peru, have reported the presence of new IMP variants, as well as the co-production of IMP-18 and VIM-2 in certain *P. aeruginosa* lineages (23).

To partially alleviate this situation, novel β-lactam compounds have been introduced into the clinical setting, such as ceftolozane/tazobactam, ceftazidime/avibactam, imipenem/relebactam, and cefiderocol. However, none of these agents is exempt from risk of resistance development. Moreover, since ceftolozane/tazobactam, ceftazidime/avibactam, and imipenem/relebactam are not effective against metallo-β-lactamases (MBLs), their use in clinical practice where those enzymes are circulating may contribute to their selection and spread (24). Conversely, cefiderocol remains effective against strains producing MBLs (25).

Given the scarcity of data on antibiotic susceptibility, including the novel agents, resistance mechanisms, and clonal epidemiology of *P. aeruginosa* in Bolivia, this study aims to provide valuable insights that could help define and guide new surveillance programs to address this challenging issue in the country.

## MATERIALS AND METHODS

### *P. aeruginosa* strains and susceptibility testing

**Fig 1:**
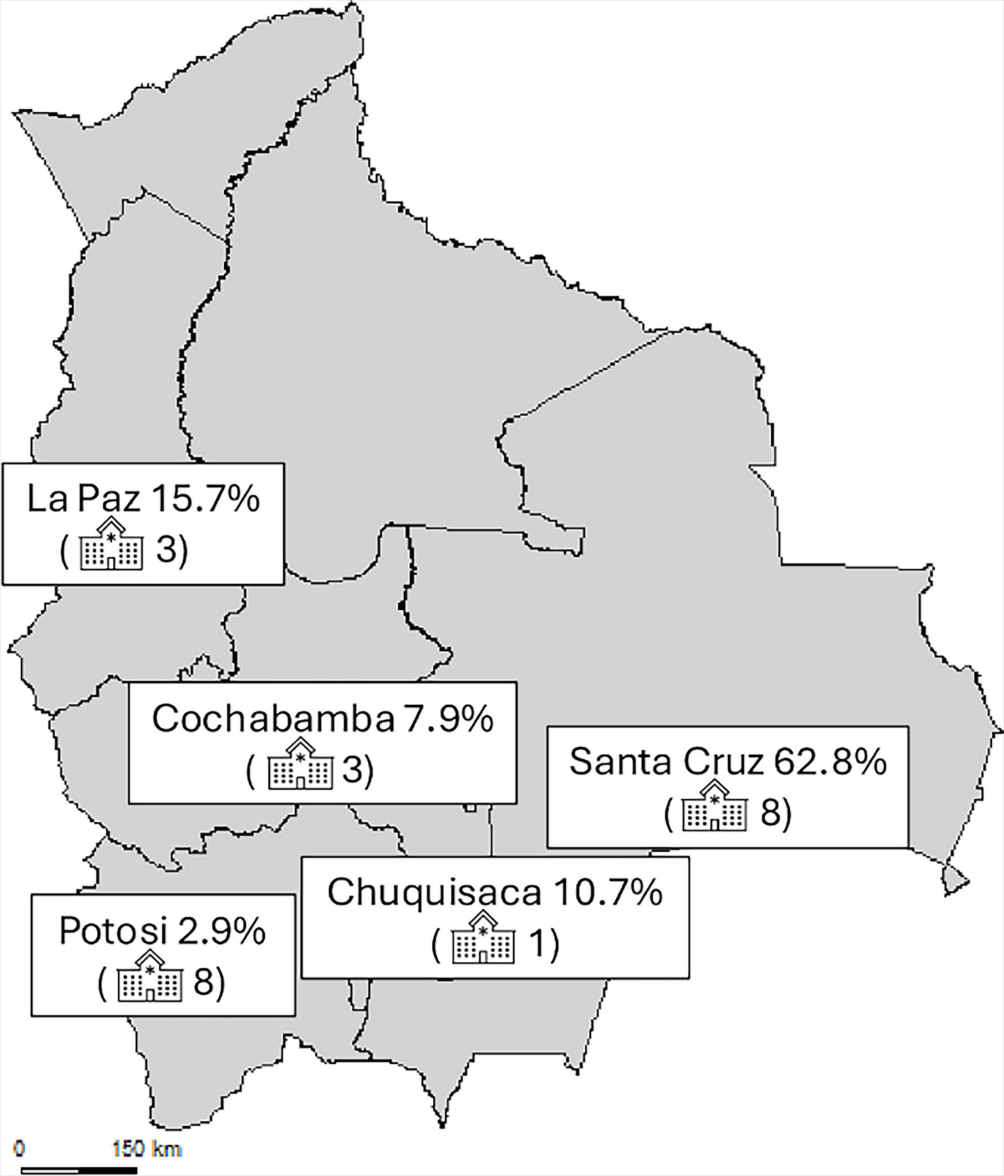
Distribution of the participating hospitals. The percentage of strains contributed to the study from each region is indicated. The number of hospitals from each of the Bolivian region is shown in parentheses. Additionally, the population covered by the participating hospitals is 1.767.783, representing 16% of Bolivia's total population.

Sixteen hospitals covering five Bolivian regions were asked to provide consecutive, nonduplicated (one per patient) XDR *P. aeruginosa* clinical isolates between May 2023 and May 2024 (Fig. 1). Sample types (respiratory, urinary, bloodstream, others) and sources (intensive care unit [ICU], medical ward, surgical ward, others) were recorded for each isolate. These isolates were identified at the UF of Bacteriology of National Center for Tropical Diseases (CENETROP, Santa Cruz, Bolivia) using standard biochemical tests and sent to Hospital Universitario Son Espases (Balearic Islands, Spain) for further characterization. The study was approved by the research committee (Ref CI-1075-25). Genospecies was confirmed using matrix-assisted laser desorption/ionization time-of-flight (MALDI-TOF) mass spectrometry (Bruker-Daltonics).

The MICs of ticarcillin (8–512 mg/L), piperacillin/tazobactam (4/4–256/4 mg/L), ceftazidime (1–64 mg/L), ceftolozane/tazobactam (0.5/4–32/4 mg/L), ceftazidime/avibactam (0.5/4–32/4 mg/L), cefepime (1–64 mg/L), imipenem (0.5–64 mg/L), meropenem (0.5–64 mg/L), aztreonam (2–256 mg/L), ciprofloxacin (0.12–16 mg/L), amikacin (2–128 mg/L), tobramycin (0.25–32 mg/L), and colistin (0.5–16 mg/L) were determined by broth microdilution using Sensititre custom panels (plate code: FRCNRP2, Thermo Fisher Diagnostics, S.LU). In addition, MICs of imipenem/relebactam (0.125–64 mg/L) were determined by in-house broth microdilution, while those of cefiderocol (0.03–128 mg/L) were determined using iron-depleted cation-adjusted Mueller-Hinton broth following the European Committee on Antimicrobial Susceptibility Testing (EUCAST) guidelines. The CLSI (M100 ED34:2024) and EUCAST 2024 (v14.0) clinical breakpoints were used for the interpretation of SIR categories. *P. aeruginosa* reference strains PAO1 and ATCC27853 and PAOΔ*piuC* (PA4515) from the PAOUW transposon library (26) were used as controls.

According to established recommendations by the ECDC and the CLSI (11), the MDR profile was defined as resistance to at least one agent in at least three of seven antibiotic classes, including antipseudomonal penicillins + β-lactamase inhibitor combinations (piperacillin/tazobactam), antipseudomonal cephalosporins (ceftazidime and cefepime), monobactams (aztreonam), antipseudomonal carbapenems (imipenem and meropenem), fluoroquinolones (ciprofloxacin), aminoglycosides (tobramycin and amikacin), and polymyxins (colistin), and the XDR profile as resistance to at least one agent in all but one or two antibiotic classes. Likewise, the PDR profile was defined as resistance to all agents in the seven antibiotic classes. DTR was defined as resistance to all first-line agents, including piperacillin/tazobactam, ceftazidime, cefepime, aztreonam, imipenem, meropenem, and ciprofloxacin (12).

### WGS

All XDR isolates were characterized through WGS.

### Library preparation

Genomic DNA was obtained with a commercially available extraction kit (High Pure PCR Template Preparation Kit, Roche Diagnostics). Indexed paired-end libraries were generated by using the Illumina DNA Prep Library Preparation Kit (Illumina, Inc., USA), and then sequenced on an Illumina Novaseq 6000 System with NovaSeq 6000 SP Reagent Kit v1.5 (300 cycles).

### Variant calling

Raw read quality was evaluated using fastQC (27). Downstream analysis showed a sequencing depth greater than 50 reads per position. The reads for each isolate were mapped against the genome of the *P. aeruginosa* reference strain PAO1 (RefSeq accession number NC_002516.2), and variant calling analysis was performed using the Snippy software (https://github.com/tseemann/snippy).

### *De novo* assembly

Reads were *de novo* assembled using SPAdes v3.15 with default options. The average size of the assembled genomes was 6.9 Mb for the analyzed strains. Assembly contiguity was assessed using standard metrics, yielding an N_50_ of 146 kb and an N_90_ of 38.7 kb, indicative of the moderate fragmentation consistent with the short-read sequencing technology. *De novo* assemblies were used to define the sequence type (ST) by using MLST v2.23.0 according to the PubMLST typing schemes (28). Additionally, assembled reads were used to study the structural integrity of the OprD porin; as different sequence variants have been described (29), the closest reference sequence was used (PAO1, LESB58, UCBP-PA14, MTB-1, FRD1, or F23197) for the variant calling analysis.

### Analysis of the mutational resistome

A total of 48 genes involved in mutational resistance were selected according to the findings of previous studies and analyzed (30–32). Nucleotide sequence variants located within these genes were filtered by using a list of natural polymorphisms that have been previously defined (33).

### Phylogenetic analysis

With the aim to study the clonal diversity of major XDR clones, core genome phylogenetic reconstruction was performed with Parsnp from the Harvest Suite package v1.2 with default parameters, forcing the inclusion of all genomes (-c) and randomly selecting the reference genome (-r!) (34). Additionally, a minimum-spanning tree (MST) for XDR strains was inferred by using GrapeTree (35) on the basis of the cgMLST scheme for *P. aeruginosa* created using the open-source ChewBBACCA algorithm (36, 37). A phylogenetic analysis, including all available IMP variants, was performed using data from the NCBI Reference Gene Catalog (https://www.ncbi.nlm.nih.gov/pathogens/refgene/). Then, multiple sequence alignments (MSAs) were performed using the online Clustal Omega tool (38), applying the mBED algorithm to construct the guide tree.

### Acquired resistance determinants

To identify possible horizontally acquired antimicrobial resistance genes, the tool ResFinder v3.1.0. with default options was used (39).

## RESULTS

### Antimicrobial resistance profiles among XDR *P. aeruginosa* isolates from Bolivian hospitals

The antimicrobial susceptibility data for a total of 140 *P*. *aeruginosa* isolates tested are shown in the supplementary material (Table S1). Upon testing in the central laboratory, 111 isolates were confirmed as XDR (110 XDR and one PDR), and 71.2% of those were also classified as DTR. Resistance rates and MIC_50/90_ for all the antibiotics tested among the 111 XDR isolates are shown in Table 1. For this set of isolates, the most active antipseudomonal agents were colistin (98.2% intermediate, MIC_50/90_ = 1/2 mg/L) and cefiderocol (92.7% susceptible, MIC_50/90_ = 0.25/4 mg/L). The activity of all other β-lactams tested was much lower, even for the combinations ceftolozane/tazobactam, ceftazidime/avibactam, and imipenem/relebactam.

**TABLE 1:** Susceptibility rates and MIC_50/90_ for all tested antibiotics in 111 XDR clinical isolates^*a*^^,^^*b*^^,^^*c*^

ATB	%S	%I	%R	MIC_50_	MIC_90_
TIC	0	0	100	512	>512
TZP	1	8	91	128	256
CAZ	9	2.7	88.3	>64	>64
C/T	16.2	1	82.9	>32	>32
CZA	20.7	–	79.3	>32	>32
FEP	6.3	13.5	80.2	>64	>64
ATM	24.3	21.6	54.1	32	128
IPM	0	1	99	>64	>64
I/R	27	1.8	71.2	32	>64
MEM	1.8	4.5	93.7	>64	>64
CIP	0	0	100	>16	>16
AMK	10.8	3.6	85.6	128	>128
TOB	1	0	99	>32	>32
CST	–	98.2	1.8	1	2
FDC	92.7	6.3	0.9	0.25	4

^*a*^ Percentage of isolates that are S (susceptible), I (intermediate), or R (resistant) out of the total (*n* = 111) for each of the tested antibiotics. CLSI (M100 ED34:2024) was used to determine the clinical breakpoints.

^*b*^ Minimum inhibitory concentrations (MICs) are represented in μg/mL. TIC: ticarcillin, TZP: piperacillin/tazobactam, CAZ: ceftazidime, C/T: ceftolozane/tazobactam, CZA: ceftazidime/avibactam, FEP: cefepime, ATM: aztreonam, IPM: imipenem, I/R: imipenem/relebactam, MEM: meropenem, CIP: ciprofloxacin, AMK: amikacin, TOB: tobramycin, CST: colistin, and FDC: cefiderocol.

^*c*^ Antibiotics without an I breakpoint defined by CLSI are indicated with a dash (–).

### Molecular epidemiology and phylogeny of XDR *P. aeruginosa* in Bolivia: impact of high-risk clones

**Fig 2:**
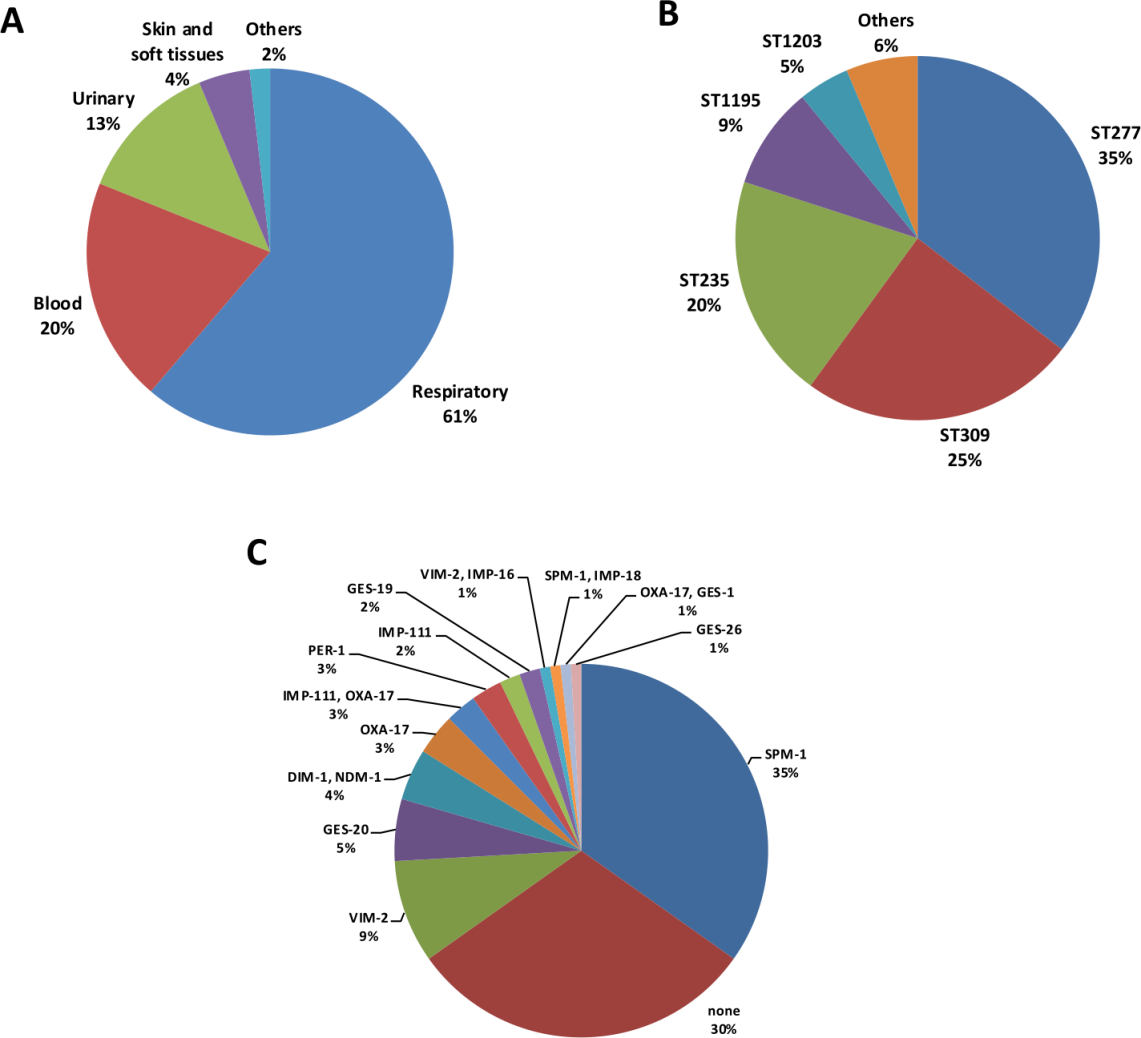
(**A**) Distribution of isolates according to the sample type. (**B**) Distribution of STs among the 140 *P*. *aeruginosa* isolates from Bolivian hospitals. (**C**) Prevalence of ESBLs and carbapenemases among XDR isolates.

**Fig 3:**
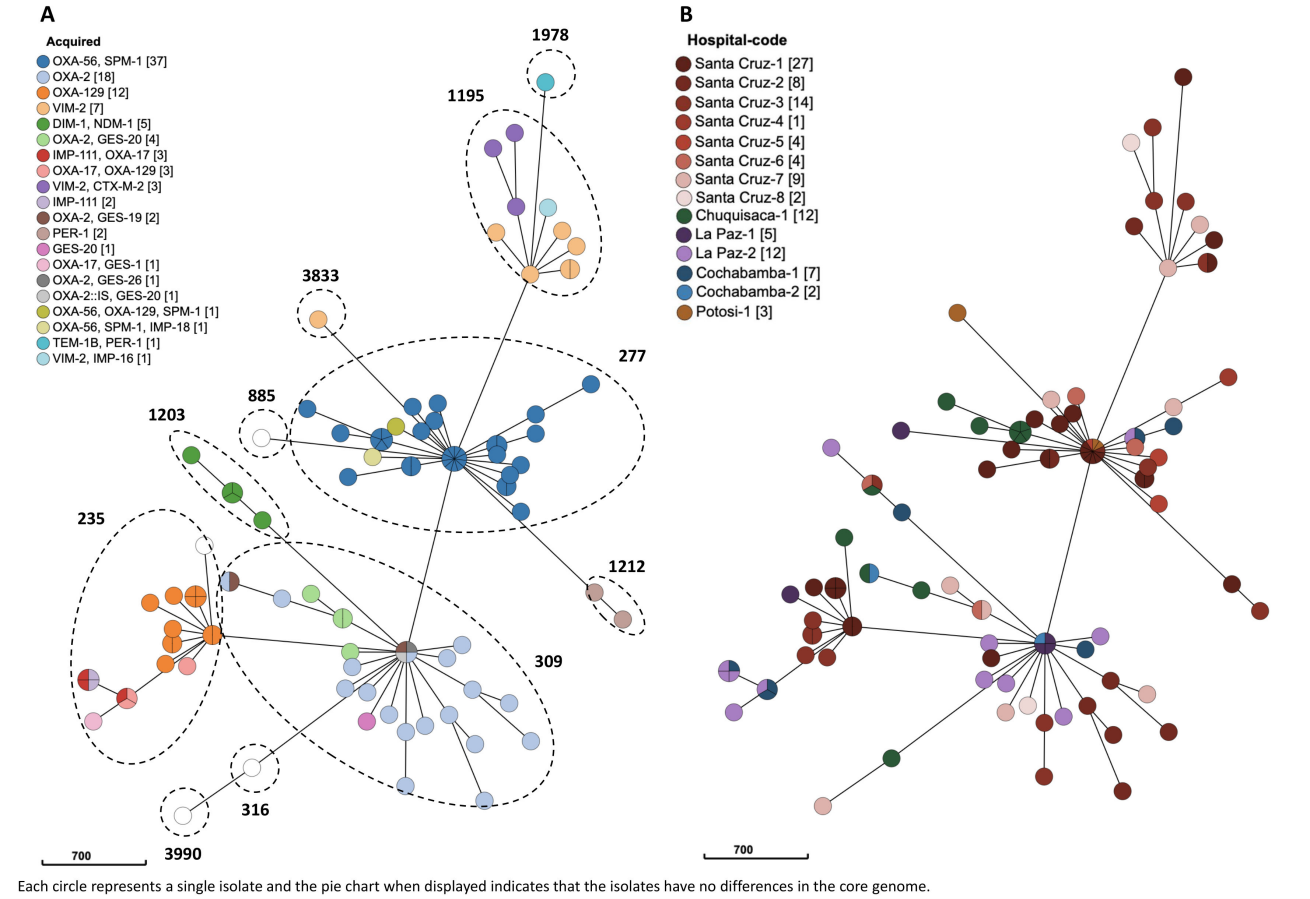
Minimum-spanning tree (MST) of the 111 XDR/PDR *P. aeruginosa* isolates based on the cgMLST profile.

Respiratory (64%) and blood (20%) samples were the most frequent, whereas urinary samples represented 13% of all isolates tested (Fig. 2A). The most frequent high-risk clone was ST277 (35%) detected in 11 different hospitals from the five Bolivian regions, followed by ST309 (25%, 11 hospitals, four regions), ST235 (20%, six hospitals, five regions), and ST1195 (9%, five hospitals, one region). Other clones detected were ST316, ST885, ST1203, ST1212, ST1978, ST3833, and ST3990 (representing from 0.8 up to 1.9%) (Fig. 2B). In addition, the cgMLST analysis of the XDR isolates indicates the presence of three main nodes corresponding to ST277, ST309, and ST235. Moreover, other smaller and well-differentiated clusters include ST1195 and ST1203 (Fig. 3A). For these major clones, both intra- and interhospital disseminations seem likely (Fig. 3B).

### Prevalence of ESBL- and carbapenemase-producing *P. aeruginosa* in Bolivia

Up to 77 of the 111 XDR isolates (70%) produced (or co-produced) an acquired carbapenemase or ESBL (Fig. 2C). Regarding these enzymes, MBLs were the most frequent, particularly SPM-1 (35%). VIM-2 and GES-20 were also found, which were detected in 9 and 5% of the isolates, respectively. Of note is the finding of a novel variant IMP-111 (PV055916), apparently representing a new cluster with the closest identity (92.35%) with IMP-105 (Fig. S1). Also noteworthy is that five isolates ascribed to ST1203 co-produced two MBLs, DIM-1 and NDM-1. Finally, the most frequently detected ESBL was OXA-17, followed by PER-1 and GES-19.

All the isolates belonging to ST277 produced SPM-1 despite being isolated in 11 different hospitals from five different regions of Bolivia. Most of the isolates that were ascribed to the ST309 produced OXA-2, and five of them also produced GES-20, three of them GES-19, and one GES-26. This clone was detected in 12 different hospitals from three different regions. The OXA-10-derived ESBL, OXA-17, was found in four isolates belonging to the high-risk clone ST235 in two different hospitals from two different regions (intra and interregional disseminations).

### Resistome and antimicrobial susceptibility profiles of the most widespread clones in Bolivia

**Fig 4:**
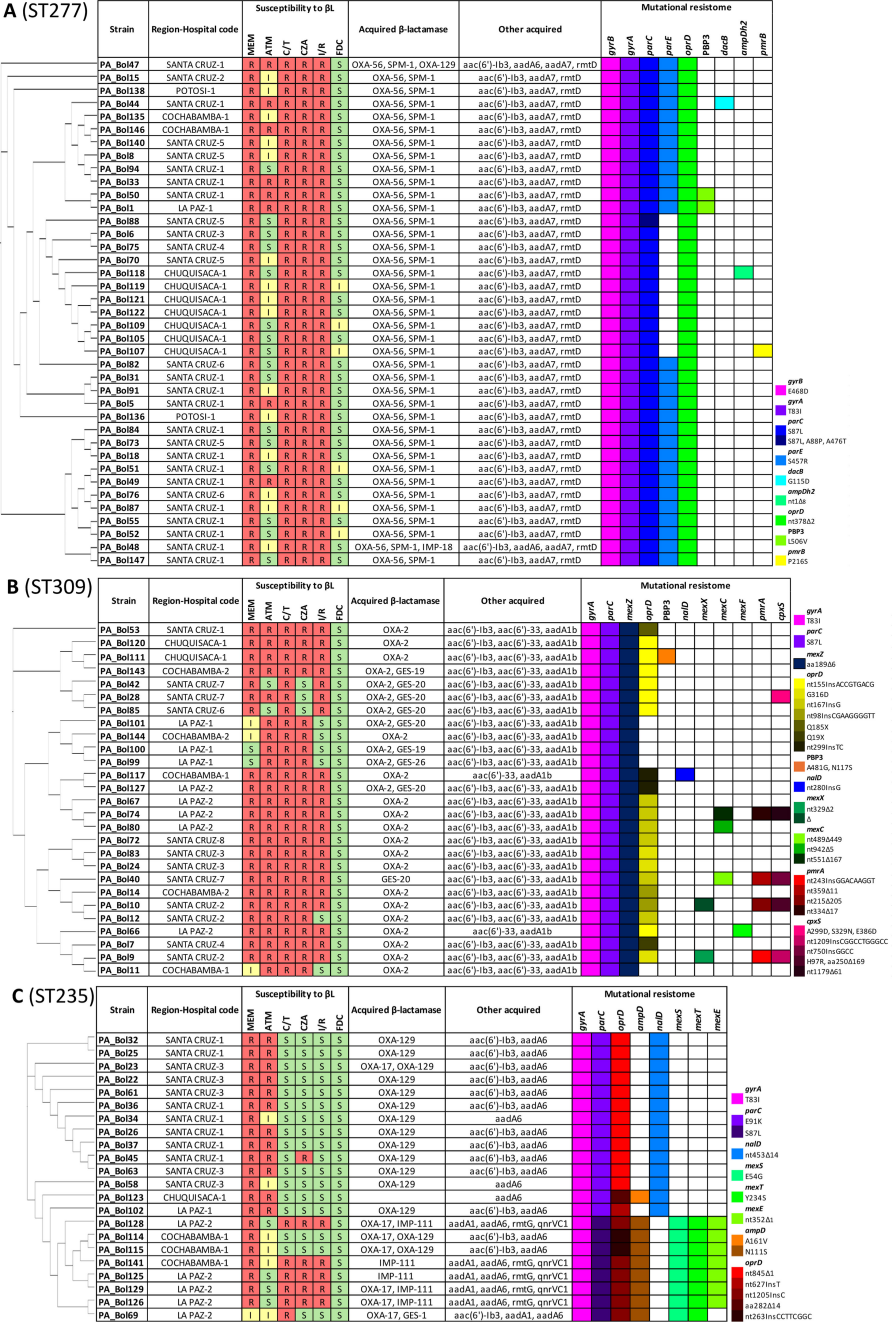
Core genome phylogenetic reconstruction of the (**A**) ST277, (**B**) ST309, and (**C**) ST235 *P. aeruginosa* isolates. Region-hospital code column corresponds to the region and the hospital within this region. Following columns correspond to susceptibility to most comon β-lactams (including novel combinations), acquired β-lactamases and other acquired enzymes. Code description of changes in most mutated genes per each ST is represented on hand-site. Each color of each column corresponds to a single mutation.

All the strains belonging to the ST277 showed high levels of resistance to novel ß-lactams (ceftolozane/tazobactam, ceftazidime/avibactam, and imipenem/relebactam), classical antipseudomonal carbapenems (imipenem and meropenem), and only moderate activity against the monobactam aztreonam (Fig. 4A). Conversely, cefiderocol remained susceptible in most of the isolates, but none was resistant. Moreover, this clone was linked to the co-production of the MBL SPM-1 and the narrow-spectrum beta-lactamase OXA-56 in addition to the 16S rRNA methyltransferase RmtD along with aminoglycoside modifying-enzymes Aac(6′)-lb3 and AadA7, thus explaining resistance to these agents (Fig. 4A). All ST277 isolates harbored the common mutations in QRDR of GyrA (T83I) and ParC (S87L) conferring resistance to fluoroquinolones. In addition, all of them had a frameshift in OprD (nt_378_∆2), thus further contributing to carbapenem resistance. Mutations in *ftsI* (PBP3), *dacB* (PBP4), *ampDh2*, and/or *pmrB* were detected in some isolates (Fig. 4A).

All the isolates belonging to the ST309 were resistant to ceftolozane/tazobactam and showed high resistance levels to the combinations ceftazidime/avibactam and imipenem/relebactam, as well as for classical antipseudomonals meropenem and aztreonam (Fig. 4B). In contrast, all the isolates were susceptible to cefiderocol. Moreover, almost all isolates belonging to this clone exhibited the OXA-2 enzyme. Two co-produced cephalosporinase GES-19, one GES-26, and six harbored the GES-20 carbapenemase variant (Fig. 4B) in addition to the aminoglycoside-modifying enzymes Aac(6′)-lb3, Aac(6′)−33, and AadA1b, as the main resistance mechanism to these agents. Additionally, all these isolates also harbored the mutations in the QRDR of GyrA (T83I) and ParC (S87L), conferring resistance to fluoroquinolones, a deletion of six amino acids in the efflux pump regulator MexZ (aa_189_∆6), and most of them had inactivating mutations in OprD due to a frameshift or premature stop codon, further increasing the resistance levels to carbapenems. Other mutations detected in resistome genes, such as *ftsI* (PBP3), *nalD*, RND efflux pump components, *pmrA*, or *cpxS*, were found in some isolates (Fig. 4B).

Susceptibility profiles of the isolates belonging to ST235 differed markedly from those of the other two most prevalent clones (Fig. 4C). Consequently, low resistance rates to ceftolozane/tazobactam, ceftazidime/avibactam, and imipenem/relebactam were observed, with approximately 29% resistance for each combination. Notably, none of the isolates was resistant to cefiderocol. In contrast, decreased susceptibility was detected for meropenem and aztreonam. Moreover, most of the ST235 isolates were associated with the narrow spectrum OXA-129, while other members also carried the ESBL OXA-17. Additionally, up to five isolates harbored the novel IMP-111 variant. ST235 isolates carried the aminoglycoside modifying enzymes AadA6, AadA1, and Aac(6′)-lb3, conferring resistance to these agents, among other resistance determinants. Also, common mutations in GyrA (T83I) and ParC (S87L, E91K) were detected for all isolates, conferring resistance to fluoroquinolones. Other mutations detected in the resistome genes were found in *oprD* (mostly inactivating), *nalD*, *mexS*, and *mexT* (Fig. 4C).

More concerning was the documented spread of the ST1203, the producer of two MBLs, DIM-1 and NDM-1, as all isolates were resistant to ceftolozane/tazobactam, ceftazidime/avibactam, imipenem, meropenem, imipenem/relebactam, and two out of five to cefiderocol. Conversely, all isolates from this clone were susceptible to aztreonam since it is stable against MBL hydrolysis and additional mechanisms, such as ESBL or mutations leading to AmpC or efflux pump overexpression, were not detected.

## DISCUSSION

This study represents the first large-scale nationwide survey in Bolivia of XDR *P. aeruginosa* clinical isolates, including the molecular epidemiology and resistance mechanisms involved. Since there are no prior studies on this topic in Bolivia, this study will set a precedent for future research on antibiotic resistance in the country.

Of particular concern is the high prevalence of resistance to novel combinations ceftolozane/tazobactam (82.9%), ceftazidime/avibactam (79.3%), and imipenem/relebactam (71.2%) detected in the present study. Previous global data have supported the *in vitro* potency of these compounds against MDR/XDR *P. aeruginosa*, with higher susceptibility rates (16, 31, 40–44). Despite the high resistance rates to these combinations, cefiderocol was active against 92.7% of the isolates, also consistent with previous studies (45); however, the detection of resistant isolates is alarming, as this antibiotic has not yet been approved for its commercial use in Latin America. Those results align with the high prevalence (up to 70%) of carbapenemases and ESBLs detected. This prevalence is higher than that found in many other areas of the world (20, 43, 46). Among those, MBLs were the most frequently detected, particularly SPM-1, aligning with data from neighboring countries, such as Chile and Brazil (47, 48), followed by VIM-2, which was first detected in Chile and Venezuela and more recently in Colombia (49). The most frequent high-risk clone detected in Bolivia was ST277. Notably, this clone is particularly endemic in Brazil (50, 51). Since its first detection in an oncology patient in São Paulo (52), this clone has played a major role in the dissemination of the SPM-1 gene across various clinical settings in the country. There are also a few reports of ST277 carrying this MBL outside of Brazil (53, 54). Moreover, the resistance mechanisms associated with this clone in the present study appear to be conserved, as SPM-1 is co-produced in all isolates with OXA-56, an OXA-10 variant (55). In addition, the resistome of this clone includes conserved mutations conferring resistance to fluoroquinolones and further resistance to carbapenems due to a frameshift mutation in OprD. Isolates from this clone were resistant to all tested antibiotics, except aztreonam, colistin, and cefiderocol, although some resistant isolates were detected. The high prevalence of this clone in Bolivia suggests its dissemination from Brazil to neighboring countries, as previously reported in Chile (47). In addition, this clone appears to be highly adapted due to its chromosomal resistome and mobile genetic elements. These features not only explain its persistence and dissemination in hospital settings but also its differentiation from ST277 isolates identified elsewhere (55).

The second most widespread clone among XDR *P. aeruginosa* isolates in Bolivia was ST309. Although this clone has not yet been widely recognized as a high-risk clone, some studies suggest its potential, as it has been detected in other South American countries, such as Brazil and Uruguay, and possesses a potent resistance machinery both acquired and intrinsic (56, 57). Most of the isolates ascribed to this clone harbored an OXA-2, and six of them co-produced with a GES-20 carbapenemase and three ESBLs (GES-19/26). The mutational resistome associated with this ST was more variable, except for those genes affecting fluoroquinolones. Isolates from this clone were resistant to all tested antibiotics, except colistin and cefiderocol.

The third most widespread clone was the high-risk clone ST235, which is the founder of the clonal complex CC235 (13). ST235 shows a worldwide dissemination, and it has been associated with over 60 different β-lactamase variants. Indeed, the association of this clone with horizontally acquired resistance determinants is overwhelming (14). Other neighboring countries, such as Brazil, have already reported the presence of this clone in their clinical isolates (58), as has Colombia (59). The most frequent acquired β-lactamase in this clone in Bolivia was the ESBL OXA-17, a derivative of OXA-10. Also, a novel variant of the IMP, IMP-111 (PV055916), was detected in five of these isolates. As evidenced previously, the mutational resistome seems not to be conserved for this clone (43). However, all members harbored QRDR mutations in GyrA and ParC. Additionally, all but one isolate showed inactivating mutations in OprD. The genes most frequently mutated in this clone include the *ampC* regulator, *ampD*, or the efflux pumps regulators, *nalD*, *mexS*, and *mexT*. Despite the XDR profile, some of the isolates ascribed to this clone were susceptible to the combinations ceftolozane/tazobactam, ceftazidime/avibactam, imipenem/relebactam, and cefiderocol.

Other clones detected were ST1195 and ST1203. It is worth noting that all ST1195 isolates were carriers of the VIM-2 MBL, as previously reported in Uruguay (60). Moreover, one of these isolates co-produced two MBLs, VIM-2 and IMP-15, a phenomenon that has also been described in Mexico (61). More concerning is the detection of ST1203 since the isolates from this clone were resistant to nearly all antibiotics tested, two of them including cefiderocol, in concordance with the presence of NDM-1 and DIM-1 in those isolates. Related to this finding, the CDC published a CRE Outbreak Evaluation Set (PRJNA288601) analysis in 2015, where three isolates from North America that were ascribed to this ST and were also producers of these two MBLs, suggested that the clonal expansion of this ST is linked to the production of these carbapenemases. The global dissemination of this clone is concerning since it has caused several outbreaks in hospitals from North America linked to the production of other ß-lactamases, such as VIM-80 and the ESBL GES-9 (62).

Thus, the analysis of the whole genome of the 111 XDR clinical isolates of *P. aeruginosa* from Bolivia has revealed the most prevalent resistance mechanisms. Fluoroquinolones’ reduced susceptibility seems to be highly conserved, as mutations in GyrA T83 residue and ParC (E91K, S87L) are present in most of the isolates. Aminoglycoside resistance is also high and mainly caused by the acquisition of aminoglycoside modifying enzymes along with emerging 16S rRNA methyltransferases. Carbapenem resistance is mostly attributed to the presence of acquired carbapenemases and/or the inactivation of OprD, which is present in 86% of the XDR isolates. Key mutations in PBP3, such as N117S and L506V, have also been detected in clinical isolates conferring resistance to the most novel ß-lactams (63). Of particular importance appears to be the L506V mutation located within the catalytic domain of PBP3 and close proximity to the KTG motif essential for transpeptidation, as it has not been previously described in clinical isolates. Thus, in combination with other resistance mechanisms, especially AmpC or MexAB overexpression, it could potentially affect a broad range of ß-lactam compounds, as it has been documented *in vitro* for other variants of this enzyme (63).

The epidemiological situation of *P. aeruginosa* in South America is characterized by high resistance rates to nearly all β-lactams, the high prevalence of high-risk clones, and the presence of multiple resistance mechanisms, particularly horizontally acquired ß-lactamases, mostly MBLs (20). Noteworthy, Bolivia’s neighboring countries, such as Chile, have already reported elevated *P. aeruginosa* resistance rates to most β-lactams, including carbapenems (64), and Peru has detected clinical isolates producing MBLs (23). The molecular epidemiology in Bolivia seems to be influenced by Brazil, as suggested by the remarkable dominance of high-risk clone ST277 (50, 65). Furthermore, ST309, which appears to be a strong candidate for consideration as a high-risk clone (56), is already circulating in several South American countries, such as Brazil and Uruguay (57), but it has also been detected in the United States and Mexico (56, 66). As expected, ST235 has been detected, therefore playing a determinant role in the global variation of the prevalence of carbapenemase-producing strains (20, 67).

Regarding novel β-lactams, resistance to ceftolozane/tazobactam, ceftazidime/avibactam, and imipenem/relebactam was very high. Concerning cefiderocol, even without prior exposure to this agent, non-susceptible strains were identified, accounting for 7% of the isolates. High-level resistance to cefiderocol (MIC = 16 mg/L) was detected in a single isolate, which was assigned to ST1203. In contrast, seven isolates exhibited an intermediate MIC (8 mg/L); one of these was also assigned to the same clone, while the others were attributed to ST277. Notably, 11 isolates showed a MIC of 4 mg/L for cefiderocol, which would be considered susceptible according to the CLSI but resistant according to the EUCAST (www.eucast.org). Resistance mechanisms to cefiderocol were linked to the detection of MBLs, primarily the co-production of NDM-1 and DIM-1 in the ST1203 isolates, and the presence of SPM-1 in the ST277 isolates. Mutations in iron transport systems known to be involved in cefiderocol resistance (68) were, however, not detected in any of the isolates. Likewise, PBP3 mutations also associated with increased cefiderocol MICs (69) were not detected in any of the cefiderocol nonsusceptible isolates. However, one of the isolates showing borderline MIC (4 mg/L) presented the above-commented L506V mutation. Also concerning was the detection of two (1.8%) resistant isolates to the last-line antimicrobial agent colistin. Both isolates belonged to the ST277. This finding is slightly higher than what has been reported in other studies from Latin America (70).

This study has some limitations. First, although the study was prospective and included the collection of consecutive non-duplicated XDR isolates, bias in recruitment success in the different hospitals cannot be fully ruled out. Moreover, some regions (e.g., Santa Cruz) seem to be overrepresented in the collection of isolates studied. Finally, valuable clinical data, including treatments administered and outcomes, were not available.

In summary, the concerning findings of this study highlight the urgent need for continuous epidemiological surveillance in Bolivia. Countries with limited access to antibiotics and essential healthcare services experience the highest age-standardized mortality rates associated with and attributable to antimicrobial resistance, emphasizing the necessity for targeted policy interventions. Therefore, it is crucial to establish appropriate antimicrobial stewardship programs and implement efficient infection control measures to minimize the emergence and spread of antimicrobial-resistant infections, particularly those caused by XDR *P. aeruginosa*. Collaborative, multisectoral efforts among regions will be essential for effectively addressing AMR in Bolivia.

## Data Availability

Genomic sequences have been deposited in the European Nucleotide Archive under project number PRJEB88506.

## SUPPLEMENTAL MATERIAL

Data S1 aac.01163-25-s0001.xlsx Characteristics of the 140 isolates fully sequenced (110 XDR, one PDR), including the sample type, source, susceptibility profiles, sequence type, acquired resistance determinants, and mutational resistome.

Fig. S1 aac.01163-25-s0002.tiff Phylogenetic tree of the 103 blaIMP variants, including the novel blaIMP-111.

ASM does not own the copyrights to Supplemental Material that may be linked to, or accessed through, an article. The authors have granted ASM a non-exclusive, world-wide license to publish the Supplemental Material files. Please contact the corresponding author directly for reuse.
