# Supplementary figures and images for: First nationwide survey on Pseudomonas aeruginosa in Bolivia: susceptibility profiles, resistome, and genomic epidemiology
Source: Antimicrob Agents Chemother. 2025 Nov 11;69(12):e01163-25. doi: 10.1128/aac.01163-25 (PMC12691617; doi:10.1128/aac.01163-25)

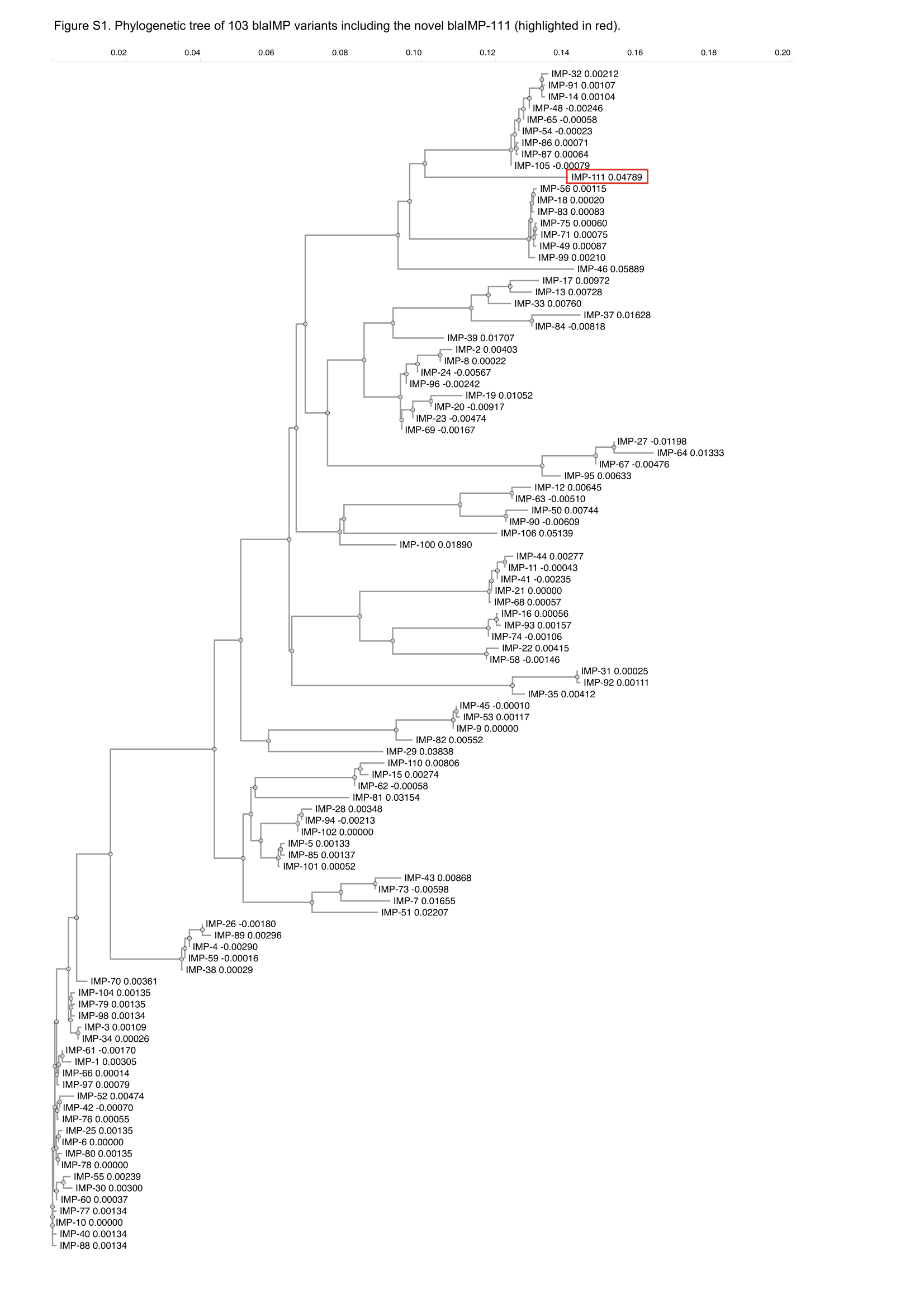

Supplement: Fig. S1 — Phylogenetic tree of the 103 blaIMP variants, including the novel blaIMP-111. [file aac.01163-25-s0002.tiff]
